# The Visuospatial and Sensorimotor Functions of Posterior Parietal Cortex in Drawing Tasks: A Review

**DOI:** 10.3389/fnagi.2021.717002

**Published:** 2021-10-14

**Authors:** Shuwei Bai, Wenyan Liu, Yangtai Guan

**Affiliations:** ^1^Department of Neurology, The Second Affiliated Hospital of Xinjiang Medical University, Urumqi, China; ^2^Department of Neurology, Renji Hospital, Shanghai Jiaotong University Medical School, Shanghai, China

**Keywords:** drawing, posterior parietal cortex, sensorimotor integration, visuospatial abilities, dementia, constructional apraxia

## Abstract

Drawing is a comprehensive skill that primarily involves visuospatial processing, eye-hand coordination, and other higher-order cognitive functions. Various drawing tasks are widely used to assess brain function. The neuropsychological basis of drawing is extremely sophisticated. Previous work has addressed the critical role of the posterior parietal cortex (PPC) in drawing, but the specific functions of the PPC in drawing remain unclear. Functional magnetic resonance imaging and electrophysiological studies found that drawing activates the PPC. Lesion-symptom mapping studies have shown an association between PPC injury and drawing deficits in patients with global and focal cerebral pathology. These findings depicted a core framework of the fronto-parietal network in drawing tasks. Here, we review neuroimaging and electrophysiological studies applying drawing paradigms and discuss the specific functions of the PPC in visuospatial and sensorimotor aspects. Ultimately, we proposed a hypothetical model based on the dorsal stream. It demonstrates the organization of a PPC-centered network for drawing and provides systematic insights into drawing for future neuropsychological research.

## Drawing Tasks

Drawing is a unique high-order human ability that transforms mental representations into fine hand movements (La Femina et al., 2009; McCrea, 2014). Drawing tasks are widely used in the clinical assessment of brain function for their easy availability and high efficiency. Performing drawing tests requires only a pen and a piece of paper, but the drawing performance yields a wealth of information on the cognitive abilities of the drawer. By evaluating the drawing performance of patients, neurologists detect cerebral injuries (Gainotti and Trojano, 2018; Rusconi, 2018), make the diagnosis of dementia (Tan et al., 2015; Salimi et al., 2018), discriminate easily confused diseases (Tan et al., 2015; Salimi et al., 2019), and predict the development of cognitive decline (Youn et al., 2021). Recently, the value of drawing tasks has attracted much attention for their sensitivity in detecting visuospatial symptoms, which are identified as early diagnostic biomarkers for Alzheimer’s disease (AD) and Parkinson’s disease (PD; Mandal et al., 2012; Zhu et al., 2020; Aarsland et al., 2021; Robinson et al., 2021).

**Figure 1:**
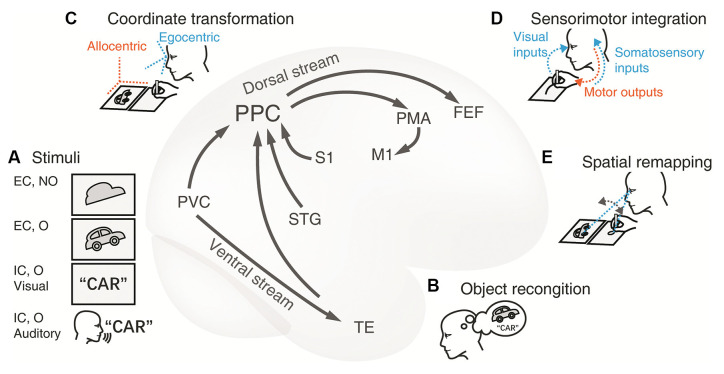
Cortical neural circuitry underlying the visuospatial and sensorimotor functions of PPC in drawing tasks. **(A) Stimuli.** The first card with an irregular shape is an externally-cued nonobject stimulus and the contour of a car in the second card represents an external objective cue. The card with a word and the auditory instructions are instances of the internal cue of familiar objects. The primary visual cortex (PVC) and the superior temporal gyrus (STG) process the visual and auditory stimuli respectively. The information is subsequently conveyed to the adjacent PPC. **(B)** **Object recognition.** Transfer of object visual information to the temporal lobe through the ventral stream. The inferior temporal lobe is recruited to recognize and name the object presented. This conceptual information is then passed to the PPC through the connections between the dorsal and ventral streams. This extra procedure facilitates the construction of graphical representation to be drawn. **(C) Coordinate transformation.** The movement of limbs is encoded within the egocentric coordination, thus, the visual representation from the allocentric (world-centered) coordinate frame is transformed to an egocentric one (e.g., eye-centered or body-centered) in the PPC.** (D) Sensorimotor integration.**The PPC integrates multidimensional inputs of vision and somatosensory from the PVC and the primary somatosensory cortex (S1) respectively. These sensory inputs together determine the current position of the limb and provide guidance for planning and adjusting the trajectory toward the target on the canvas in the PPC. Then the premotor area (PMA) encodes the motor commands, and the primary motor cortex (M1) programs the motor signals and projects to the limb. As a result, the hand/pen moves to shape the final figure on the paper. **(E) Spatial remapping.** The PPC is communicated to the frontal eye fields (FEE) during saccades, which ensures the consistency of the graphical representations between the model and the copy. Abbreviations: EC, external cue; FEF, frontal eye fields; IC, internal cue; M1, primary motor cortex; MTL, medial temporal lobe; NO, nonobject; O, object; PMA, premotor area; PPC, posterior parietal cortex; PVC, primary visual cortex; S1, primary somatosensory cortex; STG, superior temporal gyrus; TE, rostral inferior temporal cortex.

Drawing tasks can be classified into externally-cued (e.g., copying from an existing model) and internally-cued drawings (e.g., drawing from memory and imagery) according to the stimuli (Yuan and Brown, 2014, 2015; Griffith and Bingman, 2020). Moreover, drawing a familiar object (objective drawing) is distinguished from drawing unfamiliar or meaningless stimuli (nonobjective drawing; Yuan and Brown, 2015; Griffith and Bingman, 2020; Raimo et al., 2021). In addition, the need for creativity, complexity of stimuli, and other attributes should also be considered when performing drawing tasks (see Table 1, Figure 1A; Saggar et al., 2017).

**Table 1:** Comparison of common clinical drawing tests.

Drawing tests	Stimuli	Symmetry of the stimuli	Elements of the stimuli
MMSE-PCT (Folstein et al., 1975)	EC, NO	Bilateral	Pentagons
MoCA-CDT (Nasreddine et al., 2005)	IC, O	Central	Circle, lines, and numbers
MoCA-Cube copying (Nasreddine et al., 2005)	EC, O	Central	Squares and parallelogram
ROCFC (Shin et al., 2006)	EC and IC, NO	None	Multiple regular geometric figures
Human face copying (Schaer et al., 2012)	EC, O	Bilateral	Curves and irregular geometric figures
Torrance Tests of Creative Thinking (Torrance, 1972)	IC, NO/O	Unrestricted	Geometrical figures

*Abbreviations: CDT, clock drawing test; EC, externally-cued drawing; IC, internally-cued drawing; MMSE, Mini-mental state examination; MoCA, Montreal Cognitive Assessment; NO, nonobjective; O, objective; PCT, pentagon copying test; ROCFC, Rey-Osterrieth complex figure copying*.

To interpret the neural substrates of drawing, several theoretical neuropsychological models have been developed (Roncato et al., 1987; Sommers, 1989; Grossi, 1991; La Femina et al., 2009; McCrea, 2014). One of the most accepted cognitive models of drawing proposed by Sommers et al. posited that drawing mainly relies on visual perception and graphic production systems (Sommers, 1989; Guérin et al., 1999). Additionally, Roncato et al. (1987) presumed four stages in the externally-cued drawing: exploring the model, preparing the drawing plane, executing the drawing plan, and comparing the drawing to the model. La Femina et al. (2009) organized the drawing procedure into preliminary analysis, preparation of drawing plan, execution, and control processes. From the above theories, it can be concluded that visuospatial encoding of visual representations (visuospatial function) and execution of sensory-guided movements (sensorimotor function) are two fundamental components involved in drawing (McCrea, 2014). Certainly, other cognitive domains such as lexical semantics, visual imagination, and memory processes, may be engaged under specific drawing circumstances (Roncato et al., 1987; Trojano et al., 2009; Paula et al., 2013; Senese et al., 2015; Trojano and Gainotti, 2016).

Visuospatial abilities include the intelligence to specify the parts and overall configuration of a percept, appreciate its position in space, integrate a coherent spatial framework, and perform mental operations on spatial concepts (Salimi et al., 2018). In drawing situations, visuospatial processing produces mental images drawn from the stimuli, which are subsequently transformed into limb movements. Sensorimotor integration is the ability to incorporate sensory inputs from the body and the environment to inform and shape motor output (Edwards et al., 2019). In drawing tasks, sensory inputs provide information about the position of the hand and guide the hand to reach the target loci on canvas. The posterior parietal cortex (PPC) plays a critical role in visuospatial (Whitlock, 2017; Xu, 2018; Hadjidimitrakis et al., 2019) and sensorimotor functions (Chivukula et al., 2019; Edwards et al., 2019). Under the grand frame of the drawing model, here we endeavor to depict the visuospatial and sensorimotor aspects which are specified to be highly associated with the PPC in drawing tasks (Averbeck et al., 2009; Raimo et al., 2021). To better understand the functions of PPC in drawing tasks, we reviewed neuroimaging and electrophysiological studies investigating the anatomic-clinical correlates.

## The Anatomy of The PPC

The PPC comprises the superior parietal lobule (SPL), inferior parietal lobule (IPL), and intraparietal sulcus (IPS). This anatomical region can be approximately equal to the Brodmann Area 5 (BA5), BA7, BA39, and BA40 (Whitlock, 2017; Caspers and Zilles, 2018). The medial portion of the parietal lobe is the precuneus (preCun). The IPL consists of the supramarginal gyrus (SMG, BA40) and the angular gyrus (AG, BA39). The SPL and IPL are further subdivided into a mosaic of cytoarchitectonically distinct areas (Caspers and Zilles, 2018).

The PPC is one of the key association cortices in the brain. It is adjacent to the postcentral gyrus, the occipital and temporal lobes connecting the distant frontal lobe and subcortical regions through the superior longitudinal fasciculus, middle longitudinal fasciculus, and arcuate fasciculus (Caspers and Zilles, 2018).

## The Association Between Drawing and PPC

### Drawing Activates the PPC

Numerous functional magnetic resonance imaging (fMRI) and electrophysiological studies have shown that drawing tasks activate the PPC (see Table 2). Activation likelihood estimation (ALE) research on fMRI has identified the specific role of IPL and preCun in the core fronto-parietal network by drawing (Raimo et al., 2021).

**Table 2:** The activation of the PPC in drawing tests from fMRI studies.

Investigators	Sample size	Drawing tests	Related brain areas (function/process)
Ino (Ino et al., 2003)	18	CDT	SPL, IPS, dPMA, preSMA, vPFC, precentral gyrus, and cerebellum.
Makuuchi (Makuuchi et al., 2003)	17	Object drawing and naming	SPL, IPS, SMG PostITS and vPMA (object recognition).
Ferber (Ferber et al., 2007)	20	Drawing from memory and copying	Cuneus, LG, and ACC (copying vs. drawing from memory).
Gowen and Miall (Gowen and Miall, 2007)	10	Tracing and drawing shapes	Right cerebellar crus I, preSMA, dPMC, right SPL/preCun, and left preCun (drawing vs. tracing).
Harrington (Harrington et al., 2007)	11	Drawing and writing	BA 37 (naming), BA 44 (execution and imagery of movement), BA 7 (spatial processing), BA 40 (motor attention and working memory), FEF (eye movement).
Harrington (Harrington et al., 2009)	8	Objective and nonobjective drawing	ITG, FG, aIFG, and IPL (familiar objects vs. nonobject); IFG and ITG (semantic process).
Kowatari (Kowatari et al., 2009)	20	Designing new pens	PFC-parietal Network (creativity); training exerts a direct effect on the left parietal cortex.
Miall (Miall et al., 2009)	13	Cartoon faces drawing	Lateral occipital lobe and FFA (face processing); PPC and frontal lobe (drawing from memory).
Schaer (Schaer et al., 2012)	20	Portrait drawing	FFA and higher visual cortex (face recognition); PreCun (allocentric coordinate encoding); IPS and cerebellum (feedback during motor feedback).
Ellamil (Ellamil et al., 2012)	15	Book cover designing	MTL, dlPFC, dACC (creative generation); mPFC, PCC/preCun, TPJ (creative evaluation).
Yuan and Brown (Yuan and Brown, 2014)	15	Blind drawing, copying, and visual perception	M1, SMA, cerebellum (hand movement); FEF (eye movement) V5/MT+, V3A, LO (visual motion perception); SPL, IPL, and IPS (visuomotor coupling).
Garbarini (Garbarini et al., 2014)	12	Real and imagery tasks	preSMA, PPC (bimanual coupling); right SPL (mediating spatial interference); left PPC (motor imagery).
Park (Park et al., 2015)	48	Figural Torrance Tests of Creative Thinking	bilateral ITG, left IG, left PL, right AG, PFC (creativity).
Saggar (Saggar et al., 2015)	30	Word-guessing game of Pictionary	Cerebellum, thalamus, left parietal cortex, right SFG, left PFC and paracingulate/cingulate regions.
Saggar (Saggar et al., 2017)	36	Word-guessing game of Pictionary	DLPFC, ACC/PCC, SMA, and parietal regions (executive functioning); cerebellar–frontal connectivity (spontaneous implicit).
Talwar (Talwar et al., 2019)	33	CDT	Fontal, occipital and parietal lobes; DNN negative activation.

*Abbreviations: ACC, anterior cingulate cortex; CDT, clock drawing test; dACC, dorsal anterior cingulate cortex; dlPFC, dorsolateral prefrontal cortex; dPMC, dorsal premotor cortex; DNN, default neural network; dPMA, dorsal premotor area; FEF, frontal eye field; FFA, fusiform face area; IPS, intraparietal sulcus; ITS, inferior temporal sulcus; LG, lingual gyrus; LO, lateral occipital region; M1, the primary motor cortex; mPFC, medial prefrontal cortex; PCC, posterior cingulate cortex; PFC, prefrontal cortex; preCun, precuneus; preSMA, pre-supplementary motor area; SFG, superior frontal gyrus; SMA, supplementary motor area; SMG, supramarginal gyrus; SPL, superior parietal lobe; TPJ, temporoparietal junction; V5/MT+, visual area 5/middle temporal complex; V3A, visual area 3; vPFC, ventral prefrontal cortex; vPMA, ventral premotor area*.

The intended drawing starts with the encoding of mental representations from either externally or internally-cued stimuli (McCrea, 2014). Externally-cued drawing requires the drawer to directly observe and reproduce the existing model (Tchalenko and Chris Miall, 2009; Perdreau and Cavanagh, 2015). Copying from a model activated more visual processing regions, such as the middle occipital gyrus, cuneus, and lingual gyrus, than internally-cued drawing (Ferber et al., 2007; Ogawa and Inui, 2009; Saggar et al., 2015). The information of visual perception was conveyed to the PPC, given that drawing activates the projection from the occipital cortex to the IPS (Yuan and Brown, 2014). The activation of the occipito-parietal network reflects the demand for intensive visual perception, visuospatial working memory, and attention remapping components (Ferber et al., 2007; Yuan and Brown, 2014). Given that the PPC and posterior inferior temporal sulcus (pITS, BA37) were activated when the subjects named and drew the object (Makuuchi et al., 2003; Harrington et al., 2009), it was implied that the PPC links the procedure of object recognition to the drawing processes (Ino et al., 2003; Makuuchi et al., 2003; Kravitz et al., 2011; Milner, 2017), by which the information from the ventral “what” pathway is communicated to the dorsal “how” stream.

For most internally-cued drawing tasks, the drawer usually obtains the stimuli by reading or listening to a text instruction instead of viewing graphical stimuli (Ino et al., 2003; Harrington et al., 2007, 2009; Yuan and Brown, 2014; Potgieser et al., 2015; Saggar et al., 2015). These paradigms engage lexical-semantic systems and memory retrieval to generate mental representations of the object (Gainotti et al., 1983; Ellamil et al., 2012; Coslett and Schwartz, 2018). It is supported by the fact that the left fronto-temporo-parietal network, including the temporal lobe, dorsolateral prefrontal cortex, and dorsal anterior cingulate cortex, is activated when the subjects draw a familiar object from memory (Harrington et al., 2009; Ellamil et al., 2012). The left IPL, the reading area, participates in semantic processing in the internally-cued drawing (Ellamil et al., 2012; Saggar et al., 2015; Bzdok et al., 2016; Coslett and Schwartz, 2018). Some tasks require the subjects to create or design novel objects (Chen Q. et al., 2020). Such creative drawing tasks may require elaborate mental imagery and spatial transformation. The PPC plays a causal role in mental rotation ability, which manipulates figural elements and assembles them into a whole (Hawes et al., 2019). Contrastingly, some studies found that parietal activation was relatively suppressed in the creation stage (Kowatari et al., 2009; Saggar et al., 2017) while cerebellar–prefrontal connectivity was activated in improvisation (Saggar et al., 2017). The prefrontal cortex (PFC), especially the dorsolateral prefrontal cortex (dlPFC), is essential for creativity (Chen Q. et al., 2020).

ALE analysis based on fMRI studies supported greater activation including the posterior IPS, right frontal eye field, right fusiform gyrus, and the cerebellum in copying tasks than in memory-based drawing. This indicates the need for more frequent saccades and more intensive visuospatial processing under copying conditions (Yuan and Brown, 2015). In contrast, internally-cued drawings elicit distinct activation of bilateral dlPFC, the occipital-temporal region of the ventral stream (Griffith and Bingman, 2020). The difference in activated patterns is consistent with the involvement of the dorsal and ventral pathways in different drawing paradigms.

Visuospatial encoding is followed by the production and output of limb movements. In an fMRI study (Ino et al., 2003), subjects were blindfolded and asked to draw the clock hands at a given time with their index finger. The bilateral SPL, IPS, together with the dorsal premotor area, supplementary motor area, ventral prefrontal cortex, precentral gyrus, and cerebellum were activated in this blind drawing test, suggesting the involvement of the PPC in encoding the movement of drawing. Generally, almost all paradigms that require hand-drawing have reported the activation of bilateral premotor area (BA 6), IPL (BA 40), preCun, and SPL (BA 7; Raimo et al., 2021).

To confirm that activation is associated with the intended drawing, the activation pattern during drawing was compared to that under nonmotor conditions (Harrington et al., 2007, 2009; Schaer et al., 2012; Yuan and Brown, 2014; Talwar et al., 2019; Raimo et al., 2021) and non-drawing hand movements (Ferber et al., 2007; Gowen and Miall, 2007; Ogawa and Inui, 2009; Potgieser et al., 2015; Saggar et al., 2015). Compared with nonmotor tasks, more widespread regions included the IPL (BA 40), precentral gyrus, premotor area (PMA), and supplementary motor area (SMA), and the cerebellum, were activated in drawing. Similarly, in contrast to non-drawing hand tasks, drawing recruits more areas of the PMA, SMA, and SPL (Raimo et al., 2021). These results show that PPC also contributes to planning the limb movements in addition to the frontal motor area and the cerebellum (Chivukula et al., 2019). This aligns with the idea that the IPL constructs the spatial representation while the SPL is connected with visuospatial working memory and sensorimotor processing (McCrea, 2014; Griffith and Bingman, 2020; Raimo et al., 2021). Collectively, these results addressed the core function of the fronto-parietal network in the drawing.

The apparent role of the PPC and the dorsal visual network in drawing was also demonstrated by electrophysiological evidence. High-density electroencephalogram (EEG) showed that the parietal and occipital regions were associated with event-related desynchronization (ERD) activity in the low-frequency theta/alpha range (van der Meer and van der Weel, 2017). This pattern of ERD activity could enhance the involved neurons for visual processing and sensorimotor integration, resulting in cortical activation at the macro level. The desynchronized alpha-range (8–10 Hz) and beta-range (12–30 Hz) activities were more pronounced in drawing than handwriting which may represent the stage of constructing the figure form (Ose Askvik et al., 2020).

### PPC Lesions Cause Drawing Deficits

In the early 20th century, researchers noticed connections between parietal lesions and visuospatial impairments (Balint, 1909; Strauss, 1924; Mayer-Gross, 1935). Constructional apraxia (CA) is one of the most common manifestations observed in patients with parietal injury. Noninvasive neuroimaging and electrophysiological techniques facilitate the precise mapping of brain lesions with symptoms and better understand the pathogenesis of CA. Here, we discuss the lesion-symptom relationships in patients with global or bilateral cerebral injury (e.g., AD, frontotemporal dementia, and PD, see Table 3), and focal brain injury (e.g., stroke and tumors, see Table 4), respectively.

**Table 3:** Correlations between the PPC and drawing deficits in patients with global brain injury.

Investigators	Drawing tests	Diseases	Imaging method	Related brain areas
Matsuoka (Matsuoka et al., 2011)	CDT	AD, MCI	MRI	Right parietal lobe (general); right posterior ITG, preCun, PTL, left MTG and STG (Shulman criteria); right preCun, posterior ITG (Rouleau criteria); right posterior STG (CLOX1 criteria).
Possin (Possin et al., 2011)	Benson figure copying	AD, bvFTD	MRI	PPC (AD); dlPFC (bvFTD).
Serra (Serra et al., 2014)	Figure drawing, copying	AD	MRI	BA7, BA37, BA21, BA39, BA23/31, BA18.
Barrows (Barrows et al., 2015)	CDT	AD, BvFTD	MRI	Dorsolateral frontal-parietal network (executive hand placement).
Hirjak (Hirjak et al., 2017)	CDT	AD	MRI	Bilateral temporal lobe, IPL, and right SMG.
Van der Stigchel (Van der Stigchel et al., 2018)	PCT	AD	MRI	Right parietal lobe but not frontal lobe (spatial remapping).
Zink (Zink et al., 2018)	BVMT, JoLO, BDT	Dementia, dyskinesia	MRI	Right parietal lobe (BDT); left parietal lobe (BVMT-R); Temporal lobe (JoLO).
Lee (Lee et al., 2008)	CDT	AD	PET	IPL and PCC
Takahashi (Takahashi et al., 2008)	CDT	AD	PET	Left parietal lobe, AG, bilateral hippopotamus.
Shon (Shon et al., 2013)	CDT	AD	PET	Bilateral temporoparietal lobe and left MTG (drawing from memory), bilateral temporoparietal lobe (copying).
Matsuoka (Matsuoka et al., 2013)	CDT	AD	PET	Bilateral parietal lobe, posterior temporal lobe, and right MTG (total score); bilateral parietal lobe, right posterior temporal lobe, occipital lobe, and MFG (clock hands orientation).
Melrose (Melrose et al., 2013)	ROCFC	AD	PET	Bilateral temporal-parietal cortex and occipital lobe, and right frontal lobe.
Nakashima (Nakashima et al., 2016)	CDT	AD	SPECT	BA40 (number loss), BA40, and BA7 (uneven spacing among the numbers).
Yoshii (Yoshii et al., 2018)	ADAS-Jcog	AD	SPECT	Right parietal lobe, STG, MTG, AG, and PCC.

*Abbreviations: ACE, Addenbrooke’s Cognitive Examination; AD, Alzheimer’s disease; ADAS-Jcog, Alzheimer’s Disease Assessment Scale, Cognitive Subscale (Japanese version); AG, angular gyrus; BDT, block design test; BVMT, Brief Visuospatial Memory Test-Revised Copying Trial; bvFTD, behavioral variant of frontotemporal dementia; CDT, Clock Drawing Test; CLOX1, Clock Drawing Task 1; dlPFC, dorsolateral prefrontal cortex; IPL, inferior parietal lobe; ITG, inferior temporal gyrus; JoLO, Judgment of Line Orientation test; MCI, mild cognitive impairment; MFG, middle frontal gyrus; MRI, magnet renounce imagination; MTG, middle temporal gyrus; PET, positron emission tomography; PCC, posterior cingulate cortex; PTL, posterior temporal lobe; preCun, precuneus; ROCFC, Rey-Osterrieth complex figure copying; SPECT, single-photon emission computed tomography; STG, superior temporal gyrus; VOSP, Visual Object and Space Perception*.

**Table 4:** Correlations between the PPC and drawing deficits in patients with focal brain injury.

Investigators	Drawing tests	Diseases	Hemisphere	Methods	Related brain areas
Vocat (Vocat et al., 2010)	Gainotti–Ogden figure copying	Ischemic stroke	Right	VLSM	dlPFC, PPC (hyperacute phase); dlPFC, PPC and TPJ (subacute phase).
Chechlacz (Chechlacz et al., 2014)	BCoS figure copying	Stroke	Both	VBM	Right BG, Tha (total score); right IPL, MFG (left egocentric neglect); right IG, left LG and calcarine (relative position); right AG, Put, IG (left asymmetry score); right MTG, ITG, AG, IPS, left PreCun (left asymmetry score); left calcarine, Cun, PreCun, IG, cerebellum (local features); right MTG (global features).
Chen (Chen et al., 2016)	BCoS figure copying	Ischemic stroke	Both	VLSM	Right thalamus, MFG, left IPL, postCG (high-level motor control); right MOG extending to FG, left LG, RO (visuo-motor transformation); right LG, preCun, FG, cerebellum, left IFG (interacting with objects and planning).
Toba (Toba et al., 2018)	GEREN battery	Ischemic stroke	Right	VLSM	Right AG and SMG (neglect); right AG, SLF, and IFOF (copying score).
Tranel (Tranel et al., 2008)	CDT	Multiple causes	Both	PM3	Right parietal lobe (visuospatial errors); left frontal lobe (time concept related errors).
Biesbroek (Biesbroek et al., 2014)	ROCFC, JLO	Ischemic stroke	Right	VLSM	Right frontal lobe, SMG, STG (both ROCFC and JLO); right IPL, SPL, AG, MOG (ROCFC only).
Russell (Russell et al., 2010)	ROCFC, BDT	Stroke	Right	LS	Right temporoparietal junction and IG.
Kenzie (Kenzie et al., 2015)	BIT	Stroke	Right	VLSM	SPL, IPL, STG, MTG (allocentric neglect); PreCG, MFG, IG, Cau (egocentric neglect).
Carson (Carson et al., 2019)	Star and cube copying	Stroke	Both	VLSM	Right STG, IG, RO, TP (presence of neglect); right IG, STG, MTG, SMG (left cube face omission).

*Abbreviations: AG, angular gyrus; BDT, block design test; BG, basal ganglion; BIT, Behavioral Inattention Test; Cau, caudate nucleus; dlPFC, dorsolateral prefrontal cortex; FG, fusiform gyrus; IFG, insular gyrus; IFOF, inferior fronto-occipital fasciculus; IG, insular gyrus; IPL, inferior parietal lobe; IPS, intraparietal sulcus; ITG, inferior temporal gyrus; JoLO, judgment of line orientation test; LS, lesion subtraction; MOG, middle occipital gyrus; MTG, middle temporal gyrus; PM3, lesion proportion difference map; postCG, postcentral gyrus; PPC, posterior parietal cortex; preCun, precuneus; Put, putamen; RO, rolandic operculum; ROCFC, Rey-osterrieth complex figure copying test; SLF, superior longitudinal fasciculus; SPL, superior parietal lobe; TP, temporal pole; TPJ, temporoparietal junction; VLSM, voxel-based lesion mapping*.

The volume loss of the PPC causes significant visuospatial impairment, leading to CA in drawing tests (Lehmann et al., 2011; Crutch et al., 2012, 2017). Zink et al. reported that the thickness of the left parietal cortex could predict the performance of the patient on the visuospatial memory test. In contrast, the right parietal thickness predicted the performance on a block-design test (Zink et al., 2018), indicating hemispheric dominance for visuospatial working memory and visuospatial construction. The scores of the clock drawing test (CDT) were negatively correlated with the thickness of the right PPC and preCun (Matsuoka et al., 2011), SMG, and bilateral temporal lobes (Hirjak et al., 2017) in the AD population. AD patients with CA show more severe atrophy of the right preCun and AG than those without CA (Serra et al., 2014). Specifically, it is inferred that the preCun is critical for placing the figure, the AG is involved in salient object detection and spatial attention reorientation, and the SMG is the necessity for the control of elaborate reaching movements (Karnath, 2001; Gharabaghi et al., 2006; Xu, 2018).

In addition to structural changes, hypoperfusion and decreased metabolism of PPC undermine the performance of the drawing tests. Decreased regional cerebral glucose metabolism in the right IPL and posterior cingulate cortex is associated with poor performance on the CDT in patients with AD (Lee et al., 2008). Temporal-parietal, occipital, and frontal lobes were correlated with the performance of Rey-Osterrieth complex figure copying (ROCFC; Melrose et al., 2013). Shon et al. (2013) detected metabolic activity in PPC with positron emission tomography under both memory-based drawing and model-based copying. Drawing from memory recruited the left frontal cortex in addition to the PPC, indicating greater demand for the executive ability for the task, highlighting the functional specialization of the visuospatial processing in PPC.

Compared with the neural degeneration disease which generally injures the whole brain, studies in patients with unilateral and focal lesions due to ischemic infarction or tumors can reveal the more precise causal relationship between PPC injury and CA. Voxel-based lesion-symptom mapping (LSM) is usually adopted for such analysis (Bates et al., 2003; Karnath et al., 2018). These studies strongly support the idea that damaging the PPC or interrupting the fibers that pass through the dorsal stream network leads to CA, which indicate the specific role of PPC in visuospatial perceptual and constructional processing (Table 4, Vocat et al., 2010; Chechlacz et al., 2014; Chen et al., 2016; Toba et al., 2018).

The different impaired subregions of the PPC exhibited distinct drawing errors. A clock-drawing study found that whether the clock hands were properly oriented was correlated with metabolism in the bilateral PPC, right occipital lobe, right posterior temporal lobe, and right middle frontal gyrus; whether the numbers were correctly arranged and placed on the clock face was influenced by the metabolism of the temporal lobe (Matsuoka et al., 2013). Furthermore, the number loss was attributed to hypometabolism in the right BA40 and the uneven spacing between the numbers of hypometabolism in the right BA40 and BA7 (Nakashima et al., 2016). These results support the dominance of the right PPC in spatial processing by correctly orienting and placing the figure elements. A voxel-based morphology study suggested that injury to the right PPC was associated with visuospatial errors in CDT, and left PPC dysfunction resulted in time-setting errors (Tranel et al., 2008). Biesbroek et al. (2014) compared the anatomic correlates for the complex figure copying and the judgment of line orientation (JLO) test, and found that constructional abilities rely on the integrity of the right SPL, IPL, AG, and middle occipital gyrus (MOG). In another voxel-based LSM study, Chechlacz et al. found that right AG injury was more likely to cause errors in the left part of the figure, while damage to the right AG, IPS, and left preCun were related to inaccuracy in the right part. Furthermore, the left calcarine cortex, temporoparietal junction, and insular gyrus might process detailed local elements, whereas the right MTG organized the overall framework (Chechlacz et al., 2014).

Although these findings emphasized the close correlation between PPC injury and drawing deficits, this does not mean that drawing errors specifically indicate PPC dysfunction. Poor performance in drawing tasks due to the damage of occipital, temporal, frontal lobe, and basal ganglion was also mentioned in most LSM results. Of note, some characteristics of the drawing or specific categories of errors were significantly correlated with the PPC, such as the left part errors in complex figure with the right AG (Chechlacz et al., 2014), and the orientation errors with the SMG (Nakashima et al., 2016; Van der Stigchel et al., 2018).

Electroencephalographic studies have found altered activity in patients with cerebral disease and showing difficulties in drawig. Compared with other structures, EEG slowing of the parietal cortex was associated with visuospatial dysfunction in patients with PD (Eichelberger et al., 2017). The reduction in the alpha/theta ratio of the right posterior region (Jaramillo-Jimenez et al., 2021) and parietal sigma EEG abnormalities during non-rapid eye movement sleep may be predictors of dementia (Latreille et al., 2016; Jaramillo-Jimenez et al., 2021).

## The Functions of PPC in Drawing

### Visuospatial Processing

Unerringly encoding the object to be drawn is a prerequisite for drawing accurately. An essential procedure of this step is to transform the spatial representation of the object from an allocentric (world-centered) space to an egocentric (body-centered) space (Buneo and Andersen, 2006; Ekstrom et al., 2017). This process is termed coordinated transformation. With this egocentric reference frame, the individual can manipulate the hand movements to reach the target on canvas (Jackson and Husain, 2006; Filimon, 2015; Edwards et al., 2019).

The PPC plays an important role in egocentric coordinate transformation. In nonhuman primates, the lateral and ventral intraparietal areas are important for egocentric-allocentric transformation (Cohen and Andersen, 2002; Chen et al., 2018). In humans, the PPC, especially the right PPC, encodes egocentric information during the perception and exploration of the peripersonal space (Chokron, 2003; Sherrill et al., 2015). Evidence demonstrated the activation of the IPS in blind drawing (Ino et al., 2003), tracing, and figure copying tasks (Ogawa and Inui, 2009), indicating its involvement in egocentric representation. Damage to the PPC severely disturbs the egocentric coordinate transformation, causing drawing errors (Chechlacz et al., 2014; Kenzie et al., 2015).

Spatial remapping refers to the operation that updates and integrates the selected visual information and spatial changes of objects into stable, successive visual representations during saccades or shifts of attention (Melcher and Colby, 2008; Wurtz, 2008; Pierce and Saj, 2019). In copying tests, spatial remapping is prominent, as the visual attention is frequently shifted between the model and the copy to ensure consistency. After an attentional shift, the newly acquired visual stimuli are seamlessly integrated into those stored before the saccade.

PPC is vital for spatial remapping operations (Melcher and Colby, 2008; Pierce and Saj, 2019). The neurons that encode saccades and coupling previous and current stimuli are located in the lateral IPS (LIP) of primates (Duhamel et al., 1992; Heiser and Colby, 2006; Subramanian and Colby, 2014; Mirpour and Bisley, 2016). The homologous region, SMG in humans, is specifically sensitive to detect intrasaccade orientation changes in goal-driven movements and is activated in tasks that depend on spatial remapping (Parks and Corballis, 2010; Pierce et al., 2019; Baltaretu et al., 2020). Spatial remapping impairments explain the failure of patients with CA to copy accurately, leading to disorganized, inaccurate images (Pierce and Saj, 2019; Pierce et al., 2019). Right AG atrophy is associated with spatial remapping dysfunction (Serra et al., 2014). SMG lesions lead to spatial remapping dysfunction deficits and cause errors in the shaping and orientation of the pentagons during the pentagons copying task (Van der Stigchel et al., 2018).

### Sensorimotor Integration

Intrinsically, drawing can be decomposed into a series of sensory-guided reaching movements. The shape and position of the figure are essentially determined by the location where the hand or pen reaches (Battaglia-Mayer et al., 2003; Huette et al., 2013). With the guidance of multisensory information, the target is set and the movement scheme is planned. In most conditions, visual information is the dominant form of sensory inputs. For blind drawing tests, inputs from the proprioception and the vestibular system instead guide hand movement.

The PPC coordinates the eyes and hands to modulate reaching movement (Jackson and Husain, 2006; Huette et al., 2013). Specifically, the PPC directs hand placement, adjusts velocity, and amends bias along the trajectory to the targeted loci (Buneo and Andersen, 2006; Jackson and Husain, 2006; Averbeck et al., 2009; Archambault et al., 2011; Battaglia-Mayer et al., 2015). In primates, the anterior intraparietal area (AIP) contains neurons for reaching and hand posture (Chivukula et al., 2019). Several areas have been associated with reaching movement, including the preCun, posterior IPS, occipito-parietal conjunction, superior parietal occipital cortex, and lateral IPS (Karnath and Perenin, 2005; Andersen et al., 2014; Xu, 2018).

Besides, damage to other parts of the parieto-frontal network can also affect the PPC’s connection, resulting in visuomotor incoordination (Caminiti et al., 2015; Gainotti and Trojano, 2018). Lesions in the frontal motor cortex that receive projections from PPC cause CA (Chen et al., 2016). Damage to the thalamus, caudate nuclei, and putamen, interrupts the connection between the PPC and the motor cortex, resulting in poor visuospatial construction (Chechlacz et al., 2014; Chen et al., 2016).

## A Hypothetical PPC-Centered Neural Circuitry for Drawing

According to the classic dual-stream theory, drawing is a typical task of the dorsal or “action” stream (Goodale and Milner, 1992; Freud et al., 2016; Milner, 2017). After that, Kravitz et al. (2011) further identified three branches that projected from the PPC for specific visuospatial skills: (1) the parietal-prefrontal pathway, which is related to visuospatial working memory and visual-guided eye movement; (2) the parietal-premotor pathway, which coordinates the position and movement of body parts with the peripheral environment; and (3) the parietal-medial temporal pathway for spatial navigation (Kravitz et al., 2011). Drawing is highly related to the first two branches. Caminiti et al. described a detailed processing frame for the fronto-parietal network. According to the theory, the sensorimotor functions of the PPC in drawing may encompass (1) visual guided hand movement (SPL); (2) visual guided hand-object coordination (ventral parietal-PMC pathway); and (3) direct kinetic and kinematic limb information processing (somatosensory cortex and medial IPS; Caminiti et al., 2015). Interestingly, drawing tasks just perfectly embody the integrated functions of the dorsal stream and concretize the functional organization of the occipital-parietal-frontal network.

With the anatomic-functional corrections, we propose a plausible model of cortical neural circuitry based on the dorsal visual pathway (Figure 1). First, the PPC is involved in the visuospatial processing for constructing the mental graphic representations. In drawing tasks, the stimuli can be either from an external or internal cue (Figure 1A). Distinct upstream occipital and temporal areas transmit the information to the PPC. Nonobjective visual stimuli (e.g., the first card with the picture of meaningless shape in Figure 1A) are directly processed through the occipital-parietal pathway. Objective stimuli (the second card with the picture of a car in Figure 1A) are synchronously recognized and conceptualized in the ventral pathway to facilitate visuospatial processing (Figure 1B). Non-graphic internally cued stimuli (the third card with the written word of “car” or the auditory instruction of “car” in Figure 1A) are initially comprehended by the semantic system; then, the graphic representation is either created out of nothing or retrieved from long-term memory.

Second, the PPC collects perceptual information, constructs the mental representation, and transforms it into an egocentric coordinate (Figure 1C), which is essential for producing limb movement. Meanwhile, the IPL also takes part in spatial manipulation in complex drawing tasks.

Third, the PPC encodes the drawing plan and directs the downstream motor cortex to produce and execute the intended movements (Figure 1D). Multiple sensory inputs such as visual perception and somatosensory are integrated for eye-hand and hand-object coordination. In this way, continuous visual feedback guides the hand to complete the drawing task. Additionally, the PPC interacts with the frontal eye area and coordinates the saccades, which are especially required for copying tasks (Figure 1E). This model may provide new insight into how the PPC works in the occipital-parietal-frontal network and how the PPC communicates between the dorsal and ventral streams.

## Conclusions and Future Perspectives

Drawing tasks are powerful neuropsychological assessment tools. The strategic anatomical location of the PPC and its extensive connections make it a bridge between sensory inputs and motor output. Evidence from fMRI and EEG studies has shown that PPC is activated in different drawing tests, and damage in the PPC is associated with various drawing errors, according to LSM research. These findings suggest that the PPC contributes to both visuospatial and sensorimotor processing in drawing.

As the neural mechanism involved in drawing activity is elusive and multifaceted, many unsolved questions remain. Although the PPC is highlighted in drawing activities, its functions are based on the comprehensive degree of association with other parts of the brain. The functional network for drawing may involve a large scale of networks such as the dorsal stream, execution network, attention network, and memory network (Yuan and Brown, 2015; Griffith and Bingman, 2020). How these complex functional networks are organized remains to be explored in future studies.

Recent studies have focused on the value of visuospatial assessment in the early prediction of dementia (Coughlan et al., 2018; Wang et al., 2020; Aarsland et al., 2021). For better diagnostic efficiency, progress has been made by applying artificial intelligence algorithms to evaluate drawing performance (Chen S. et al., 2020; Youn et al., 2021). It is feasible to anticipate the invention of assessing systems with higher accuracy for the diagnosis and differential diagnosis of cerebral disorders. Finally, despite some studies that have shown the benefits of drawing training in cognitive rehabilitation, drawing as a therapeutic method is still controversial in clinical practice. Further investigations are needed to interpret the therapeutic effect of drawing practice and its potential effect on promoting brain plasticity.

## Author Contributions

SB and WL are major contributors in designing the review and writing the manuscript. YG revised the manuscript. All authors contributed to the article and approved the submitted version.

## Conflict of Interest

The authors declare that the research was conducted in the absence of any commercial or financial relationships that could be construed as a potential conflict of interest.

## Publisher’s Note

All claims expressed in this article are solely those of the authors and do not necessarily represent those of their affiliated organizations, or those of the publisher, the editors and the reviewers. Any product that may be evaluated in this article, or claim that may be made by its manufacturer, is not guaranteed or endorsed by the publisher.
